# Evaluating the accuracy of hand models obtained from two 3D scanning techniques

**DOI:** 10.1038/s41598-020-68457-6

**Published:** 2020-07-17

**Authors:** Fang Yu, Lei Zeng, Ding Pan, Xinlei Sui, Juyu Tang

**Affiliations:** Orthopedic Department, Xiangya Hospital, No.87 Xiangya Road, Changsha, 410008 China

**Keywords:** Imaging techniques, Skin diseases

## Abstract

The aim of this study was to identify an efficient approach for 3D imaging of hand. The 3D photographs of hand were taken with Gemini structured-light scanning system (SL scanning) and CT scanning. The 3D photographs, average time of scanning and reconstruction were compared between these two indirect techniques. The reliability, reproducibility and accuracy were evaluated in these two indirect techniques and the direct measurement (DM). Statistical differences in the measurements were assessed by 99% probability, with clinical significance at > 0.5 mm. The Gemini structured-light scanning system established a complete and smooth 3D hand photograph with shorter scanning and reconstruction time. Reproducibility of CT scanning and SL scanning methods was better (P < 0.01, both) than the DM, but did not differ significantly from each other (P = 0.462). Of the 19 (31.58%) measurements obtained, 6 showed significant differences (P < 0.01). Significant differences were observed more often for circumference dimensions (5/9, 55.56%) than for length dimensions (1/10, 10%). Mean absolute error (AE) of the 10 subjects was very low for 3D CT (0.29 ± 0.10 mm) and SL scanning (0.30 ± 0.11 mm). Absolute percentage error (APE) was 4.69 ± 2.33% and 4.88 ± 2.22% for 3D CT and SL scanning, respectively. AE for the PIP circumference between the 3rd finger (0.58 mm) and 4th finger (0.53 mm) scan was > 0.5 mm, indicating significant difference between DM and CT scanning at the level of 99% probability. In this study, the Gemini structured-light scanning system not only successfully established a complete and smooth 3D hand photograph, but also shortened the scanning and reconstruction time. Compared to the DM, measurements obtained using the two indirect techniques did not show any statistically or clinically insignificant difference in the values of the remaining 17 of 19 measurements (89.47%). Therefore, either of the two alternative techniques could be used instead of the direct measurement method.

## Introduction

Three-dimensional(3D) reconstruction and modeling of hand morphology has been increasingly gaining importance for many purposes, including manufacturing of custom-made glove^1^, analysis of hand surface and volume^2,3,4^, and designing of hand surgery and humanoid robot hand^5,6,7^. Various methods of 3D imaging have been extensively applied in oral and maxillofacial surgery as well as plastic surgery^8–11^, because they collect surface data rapidly, non-invasively, accurately, reliably, and cost-effectively. These methods allow for the development of equally accurate 3D models by means of direct anthropometry, which is considered as the “gold standard”^12,13^.

Most of the currently available 3D scanning systems have been designed for the face or chest with 180∘–190∘ capture, i.e., 3dMDface (3dMD, USA)^14^ and DI3D (Dimensional Imaging, Scotland)^9^. In our previous study, we have successfully obtained 3D images of chest from 40 patients with pectus excavatum by using a Prime Sense 3D sensor^15^. However, the human hand is an annular stereoscopic structure with five fingers, which make it markedly different from arc-shaped structures. A full 360∘ scanning is essential for 3D reconstruction of hand. Moreover, it is difficult for an individual to keep hand still, even for a few seconds. Kau and Richmond stated that soft tissues was inherently difficult to study^16^, because they are inevitably affected by movement. Therefore, a support to hand is commonly used to keep still, i.e. the template and glass support^7,17^. However, the possibility of refraction errors of the glass support cannot be ruled out^18,19^. The plaster model instead of actual hand is another approach to avoid hand tremble during scanning. Yu et al. fixed the plaster hand on a rotatable disc^1^, and rotated 36° after each image capture. However, it was time-consuming to obtain ten images captured from different angles and combine them into an entire 3D image.

Therefore, to reconstruct the 3D hand image, it requires an accurate and convenient setup with a full 360° and rapid capture. Gemini structured light-scanning system was specially designed for hand based on the current structured light-scanning equipment. In this system, two modular units were installed on opposite sides of the spout with a PC-controller desktop to collect the images of both dorsal and palmar aspect of hand simultaneously. The projection grating and data collection could be completed within one second to minimize the error caused by movement of hand.

This study aims to identify an efficient approach for 3D photographs of hand. We obtained 3D photographs of actual hand by Gemini structured-light scanning system, and compared accuracy of two indirect hand measurement techniques(Gemini structured light scanning and CT scanning) with direct measurement integument hand features. To minimize the potential discrepancies caused by soft tissue deformation and movement associated with actual hand, we adopted the plaster hand models for measurement.

## Subjects and methods

### Subjects

The subjects included in this study were ten adults [6 males, 4 females; mean age 29.6 years, range 26–35 years, body mass index (BMI): 20.98–23.99 kg/m^2^], without any preexistent hand deformities or scars on their left hands. 10 plaster hand models of left hand were prepared in advance. Each participant was asked to hold fingers out straight with the fingers spread far apart to take a negative impression. Then, plaster was added to create the hand model. The study protocol was approved by the ethics committee of our institution. All participants provided written the informed consent and they did not receive any remuneration for participating in this study.

### Methods

#### 3D photographs of Gemini structured light-scanning

**Figure 1:**
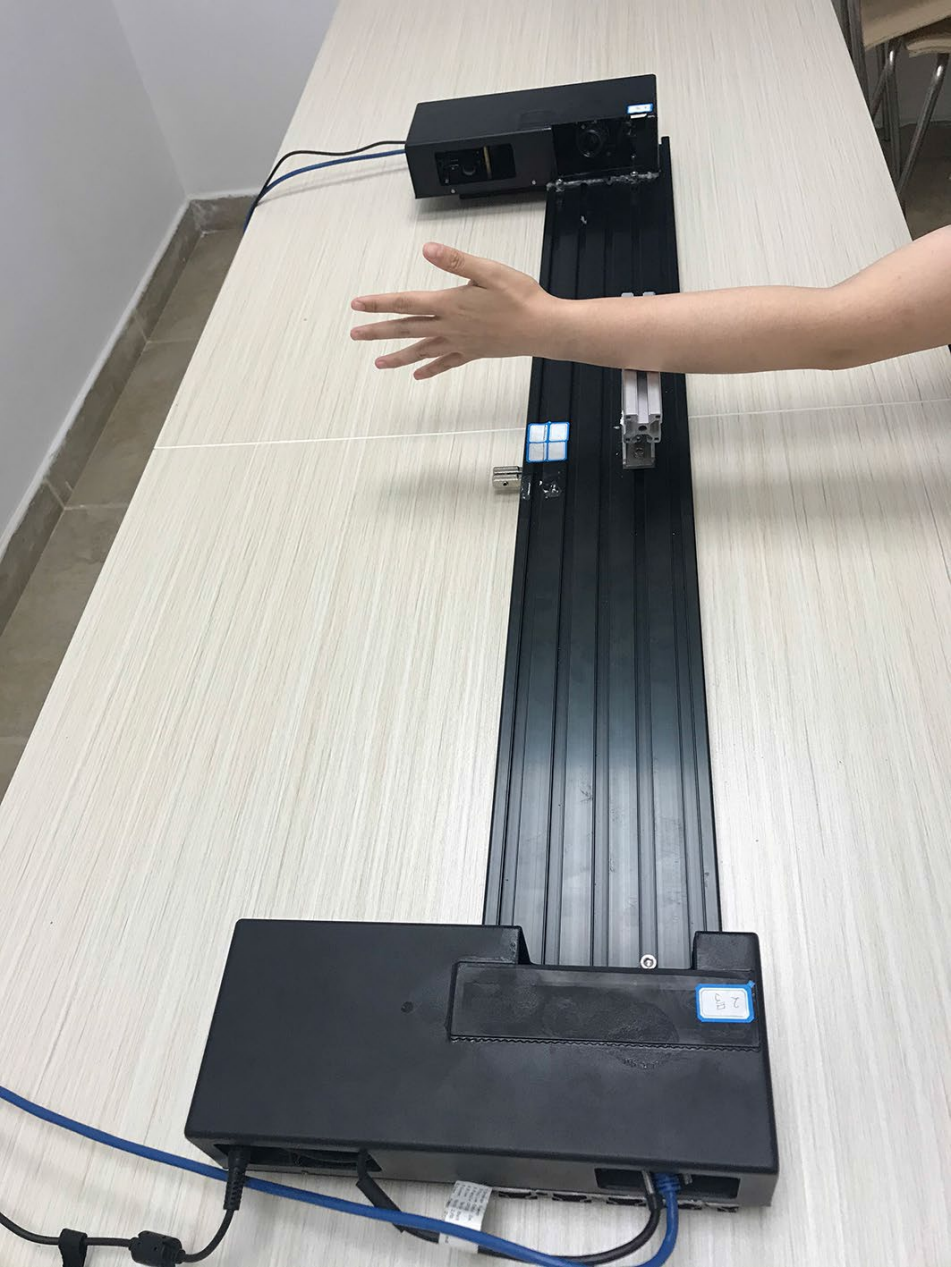
Scanning of actual hand by Gemini structured-light scanning system.

**Figure 2, 3:**
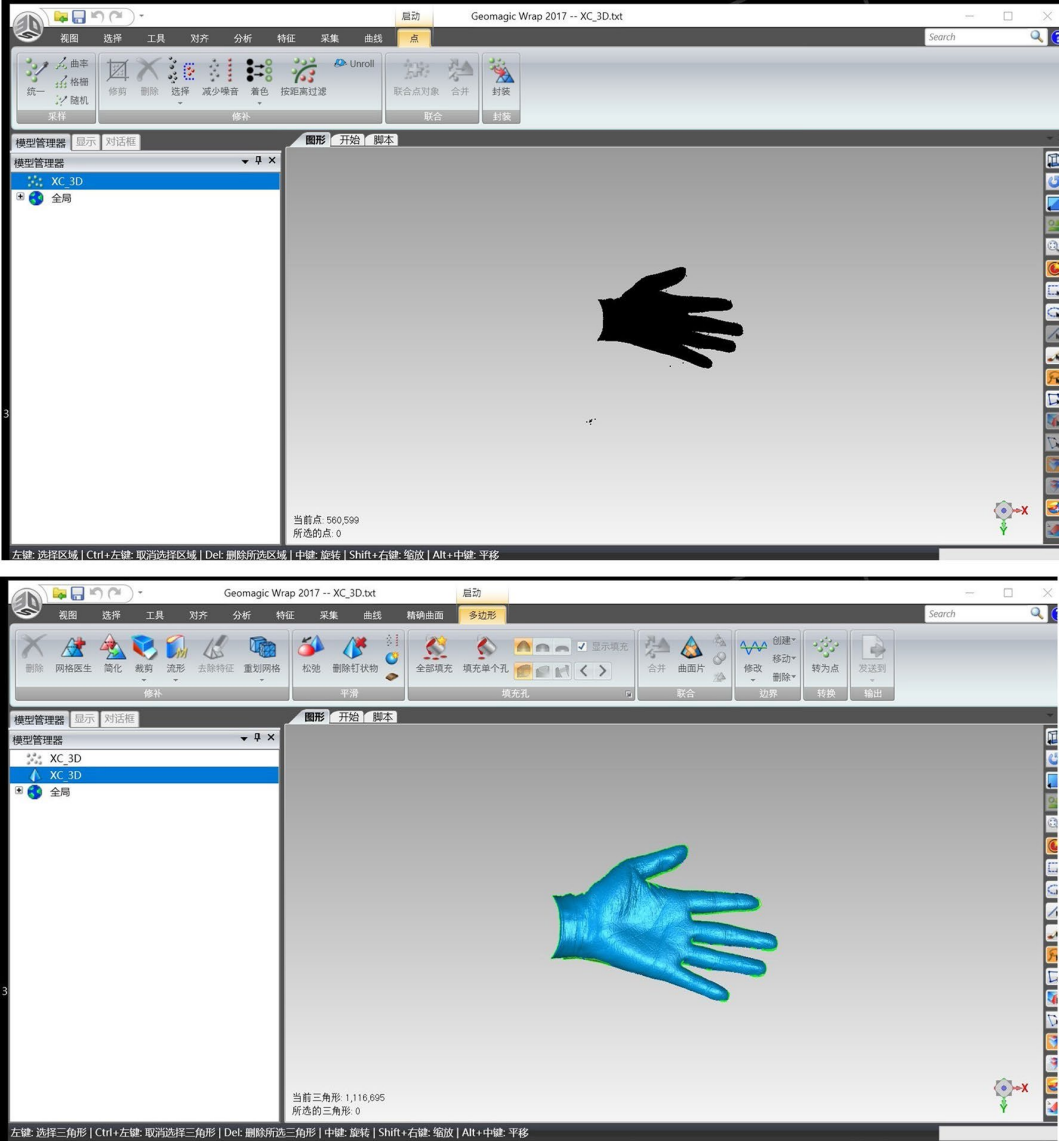
Encapsulation and filling of 3D hand model data by Wrap software.

Gemini structured-light scanning system was designed by Shenzhen Institutes of Advanced Technology, Chinese Academy of Sciences. Both modular units are equipped with a machine vision camera and industrial-grade system (with resolution of < 0.1 mm), which are synchronized in two captures. The system has a single-amplitude measurement range of 176 × 276 mm and single amplitude measurement points of 1.9 million. The projection grating and data collection could be completed within one second. The 3D photographs were taken with the subject flexion in elbow and extension in wrist joint, and the hand was kept with the fingers in a fully abducted and extended position (Fig. 1). The subject was instructed to keep the left hand absolutely still and the images of both the palmar and dorsal aspect of hand were taken. The data generated by scan was saved in the original point cloud. Wrap software (Geomagic, USA) was used to splice, encapsulate, fill, and smoothen the surface of 3D hand photograph (Figs. 2, 3), and the data was generated and saved in .STL format ultimately.

#### Measurement of 3D photographs

Measurements of each plaster hand model were obtained by three methods respectively: (1) direct measurement (DM); (2) 3D CT scanning (CT scanning); and (3) Gemini 3D structured-light scanning (SL scanning). 24 identifiable landmarks were chosen based on Hoevenaren et al.’ study of 3D hand photograph^7^ (Table 1), which has proven that intra- and inter-observer reproducibility of the most of these landmarks were high. A series of linear measurements based on the landmarks were chosen for comparative measurements (Table 2), according the standard used by Yu et al.^1^ and Hoevenaren et al.^20^.

**Table 1:** Landmarks of 3D photographs.

Landmarks	Abbreviation	Definition
D1 top	D1t	Top of 1st digital
D2 top	D2t	Top of 2nd digital
D3 top	D3t	Top of 3rd digital
D4 top	D4t	Top of 4th digital
D5 top	D5t	Top of 5th digital
IP 1 middle	IP1m	Midline of IP joint crease of thumb
DIP 2 middle	DIP2m	Midline of the 2nd DIP joint crease
DIP 3 middle	DIP3m	Midline of the 3rd DIP joint crease
DIP 4 middle	DIP4m	Midline of the 4th DIP joint crease
DIP 5 middle	DIP5m	Midline of the 5th DIP joint crease
PIP 2 middle	PIP2m	Midline of the 2nd PIP joint crease
PIP 3 middle	PIP3m	Midline of the 3rd PIP joint crease
PIP 4 middle	PIP4m	Midline of the 4th PIP joint crease
PIP 5 middle	PIP5m	Midline of the 5th PIP joint crease
MCP 1 middle	MCP1m	Midline of the 1st MCP joint crease
MCP 2 middle	MCP2m	Midline of the 2nd MCP joint crease
MCP 3 middle	MCP3m	Midline of the 3rd MCP joint crease
MCP 4 middle	MCP4m	Midline of the 4th MCP joint crease
MCP 5 middle	MCP5m	Midline of the 5th MCP joint crease
MCP 1 middle^a^	MCP1m^a^	Midline of the 1st MCP joint crease^a^
MCP 2 middle^a^	MCP2m^a^	Midline of the 2nd MCP joint crease^a^
MCP 3 middle^a^	MCP3m^a^	Midline of the 3rd MCP joint crease^a^
MCP 4 middle^a^	MCP4m^a^	Midline of the 4th MCP joint crease^a^
MCP 5 middle^a^	MCP5m^a^	Midline of the 5th MCP joint crease^a^

^a^Landmarks were on the dorsal side.

**Table 2:** Description of 19 measurements made on the hand.

Type	Measurements	Measure	Abbreviation
Circumference	IP circumference of the 1st finger	Across IP1 m	C1i
	DIP circumference of the 2nd finger	Across DIP2m	C2d
	DIP circumference of the 3rd finger	Across DIP3m	C3d
	DIP circumference of the 4th finger	Across DIP4m	C4d
	DIP circumference of the 5th finger	Across DIP5m	C5d
	PIP circumference of the 2nd finger	Across PIP2m	C2p
	PIP circumference of the 3rd finger	Across PIP3m	C3p
	PIP circumference of the 4th finger	Across PIP4m	C4p
	PIP circumference of the 5th finger	Across PIP5m	C5p
Length	1st finger length of dorsal side	D1t-MCP1m	L1a
	2nd finger length of dorsal side	D2t-MCP2m	L2a
	3rd finger length of dorsal side	D3t-MCP3m	L3a
	4th finger length of dorsal side	D4t-MCP4m	L4a
	5th finger length of dorsal side	D5t-MCP5m	L5a
	1st finger length of palmar side	D1t-MCP1m^a^	L1b
	2nd finger length of palmar side	D2t-MCP2m^a^	L2b
	3rd finger length of palmar side	D3t-MCP3m^a^	L3b
	4th finger length of palmar side	D4t-MCP4m^a^	L4b
	5th finger length of palmar side	D5t-MCP5m^a^	L5b

^a^Landmarks were on the dorsal side.

In DM, a measuring tape (with a minimum scale of 1 mm and thickness of 0.4 mm) was used for measuring the circumference dimensions and a sliding caliper (with an accuracy of 0.01 mm) was used for measuring the length dimensions. The CT scanning was constructed in the palm-up position of hand model with Neuviz 128 (Neusoft, China), and the slice thickness was 0.4 mm. The spatial resolution of X and Y axis is 0.29 mm and the Z axis is 0.32 mm. The acquired DICOM datasets were imported into Mimics Research software 19.0 (Materialise, Belgium). The 3D photographs of hand were generated and saved in the format of .STL. In SL scanning, the plaster hand model was placed in the upright position in the middle of two modular units. After processed by Wrap software, the 3D photographs were also saved in .STL format.

**Figure 4, 5:**
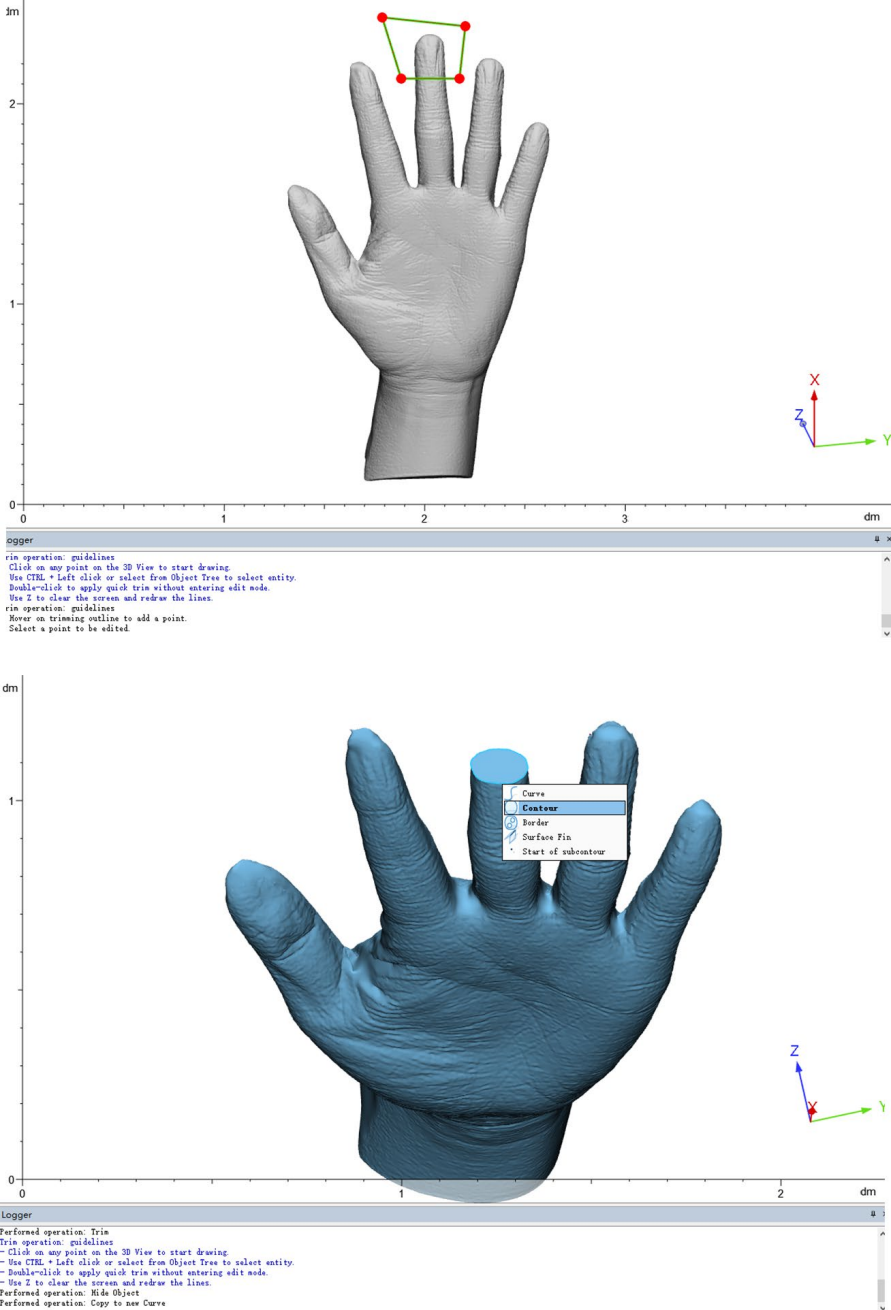
Process of circumference measurement.

In DM, the measurements were taken following ISO 7250-1:2008 standard. In SL and CT scanning methods, the 3-matic Research software (Materialise, Belgium) was used open .STL format, and measure the 3D hand model. The length dimension was the distance between two points. For circumference measurement, a horizontal surface was made crossing the middle of joint (Fig. 4), and removed the distal area with trimming method from entity (Fig. 5). The perimeter of cross section was the circumference of joint. Two weeks later, the measurements of three methods were obtained again in the same manner.

**Figure 6, 7:**
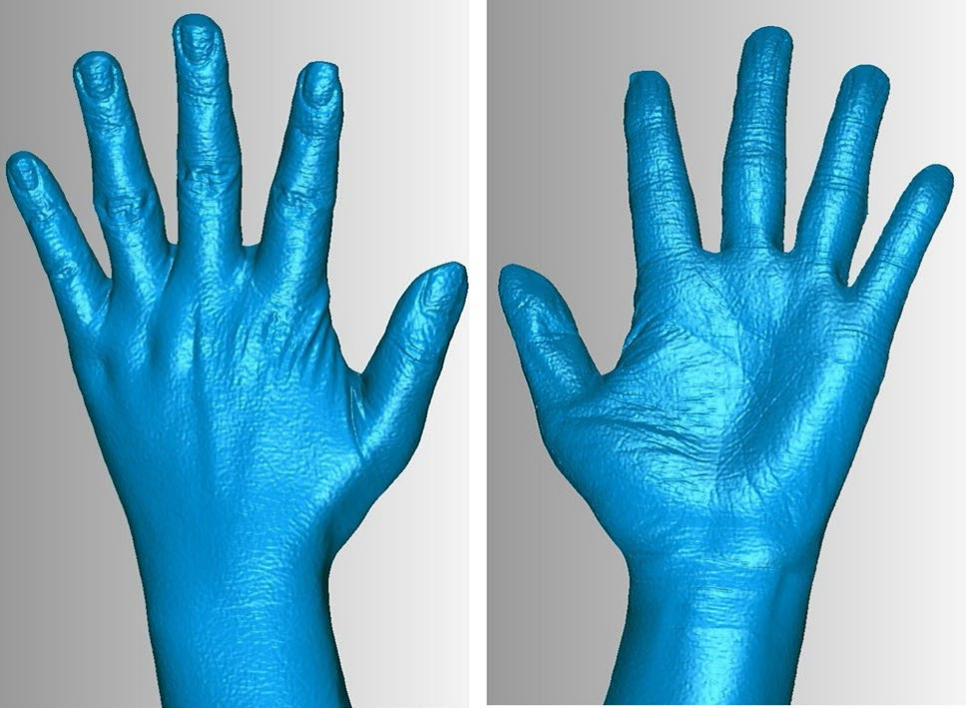
3D photographs of actual hand by SL scanning.

**Figure 8, 9:**
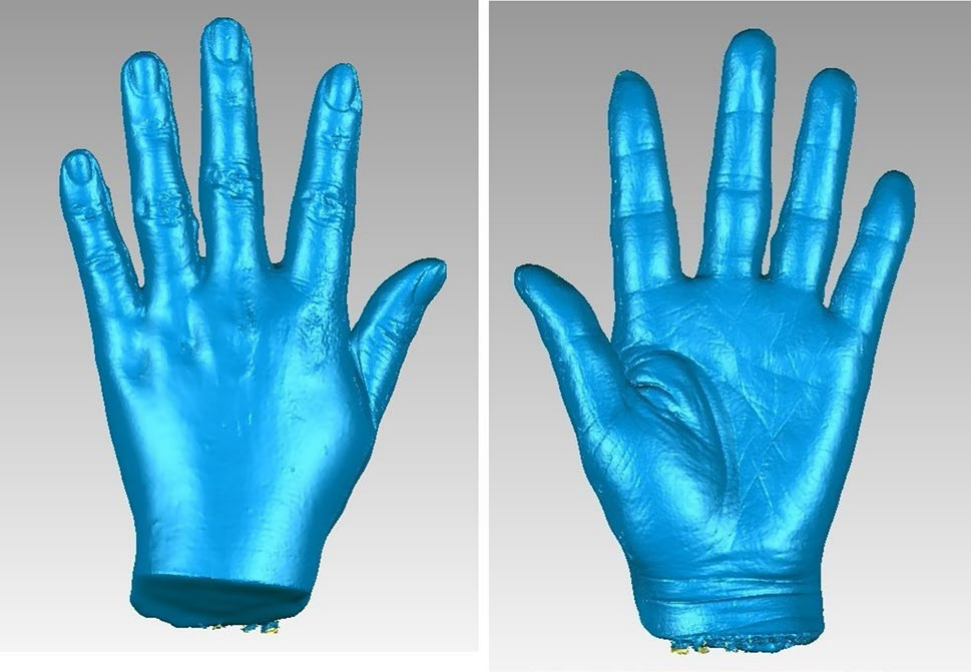
3D photographs of plaster hand model by SL scanning.

### Statistics analysis

All statistical analyses were performed with IBM SPSS Statistics, version 22 (IBM, USA). The means and standard deviations were calculated for each measuring technique. As a measure of reliability, the intraclass correlation coefficient (ICC) for absolute agreement was calculated on the basis of a two-way random effects analysis of variance (ANOVA). ICCs of the repeated measurements were used to test the reliability of three measuring techniques. The measurements obtained from CT and SL scanning were then compared with those obtained from DM by using paired *t* tests. The probability level of P < 0.01 was considered to represent statistical significance. The differences in the measurement between any of the methods greater than 0.5 mm were considered clinically significant.

The accuracy of the measurements obtained using the two 3D methods was expressed in terms of the absolute error (AE) and absolute percentage errors (APE). AE was defined as the measurement value subtracted by the reference value, which was calculated as the mean of the DM, whereas the two 3D values measurements were determined by the mean of the measurements made by the two independent examiners. APE was calculated using the following equation: APE = 100 × (AE/reference value).

### Ethical approval

All procedures performed in studies involving human participants were in accordance with the ethical standards of the ethics committee of Xiangya Hospital.

### Informed consent

Informed consent was obtained from all individual participants included in the study.

## Results

**Figure 10, 11:**
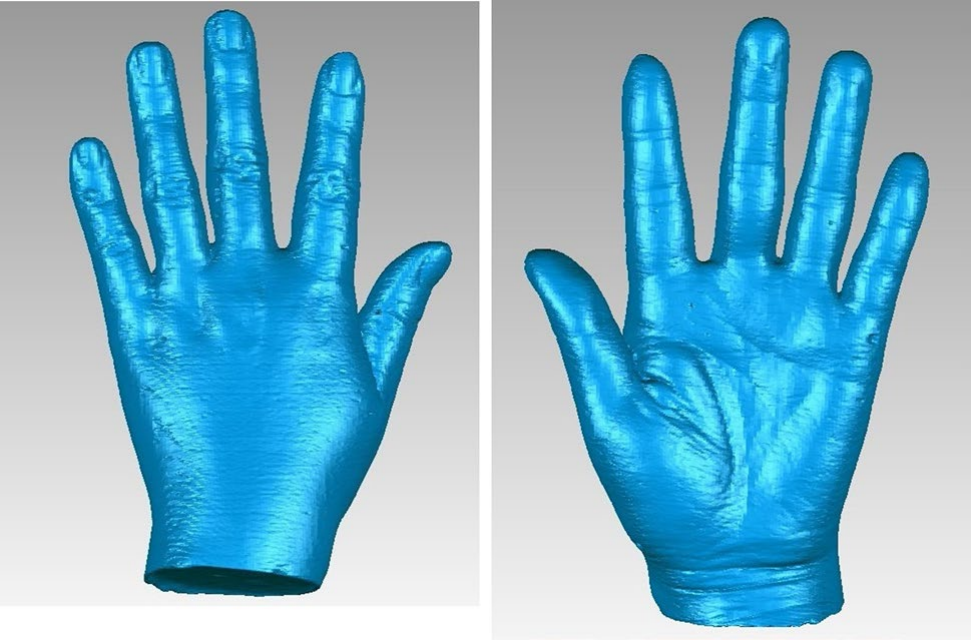
3D photographs of plaster hand model by CT scanning.

It reveals that Gemini structured light-scanning system can successfully establish a complete and smooth 3D hand photograph of actual hand (Figs. 6, 7) and plaster hand model (Figs. 8, 9). The average time of SL scanning and reconstruction of a hand model was 0.9 s and 2.4 min. In CT scanning of plaster hand model (Figs. 10, 11), the average time was 8.9 s for scanning and 6.5 min for reconstruction.

The results of DM between two observers were found to be very reliable (ICC; 0.925–0.995)(Table 3). The intra-observer reliability for CT scanning (ICC; 0.946–1.000) and SL scanning (ICC; 0.984–1.000) was also very high. Both CT and SL scanning showed higher reproducibility (P < 0.01, both) than DM. However, the two 3D scanning methods did not show any significant difference (P = 0.462).

**Table 3:** Intraclass correlation coefficients (ICC): intra-observer reliability of the three techniques.

	DM	CT scanning	SL scanning
C1i	0.974	0.991	0.985
C2d	0.940	0.999	0.998
C3d	0.968	0.999	0.993
C4d	0.939	0.997	0.994
C5d	0.940	0.999	0.985
C2p	0.963	0.986	0.994
C3p	0.995	0.946	0.985
C4p	0.990	0.975	0.991
C5p	0.925	0.994	0.984
L1a	0.978	0.996	1.000
L2a	0.987	0.999	0.997
L3a	0.933	1.000	0.994
L4a	0.983	0.998	0.999
L5a	0.945	0.997	0.998
L1b	0.936	0.965	1.000
L2b	0.987	0.999	0.998
L3b	0.976	0.999	0.999
L4b	0.955	1.000	0.994
L5b	0.985	0.999	1.000

For 6 of 19 measurements (31.58%), the significant differences were noted (P < 0.01) (Table 4). Significant differences were observed more often observed in the case of circumference dimensions (5/9, 55.56%) than in the length dimensions (1/10, 10%). In addition, CT scanning values were significantly greater for 3 of 4 (75.0%) measurements that differed; all values obtained with SL scanning were all significantly smaller than the DM.

**Table 4:** Mean and standard deviations (SD) of three anthropometric measuring techniques (all measurements in mm).

Measurements	DM		CT scanning			SL scanning		
	Mean	SD ( ±)	Mean	SD ( ±)	*P*	Mean	SD ( ±)	*P*
C1i	6.45	0.31	6.32	0.37	0.4248	6.05	0.43	0.0384
C2d	5.30	0.29	5.08	0.36	0.1663	4.82	0.33	0.0043
C3d	5.32	0.32	5.08	0.33	0.1289	4.85	0.39	0.0112
C4d	4.39	0.21	4.80	0.27	0.0020	4.58	0.28	0.1264
C5d	4.12	0.21	4.47	0.27	0.0067	4.25	0.25	0.2489
C2p	6.01	0.37	6.12	0.32	0.5024	5.91	0.44	0.6017
C3p	6.32	0.41	6.33	0.43	0.9644	6.22	0.35	0.6040
C4p	5.47	0.36	5.97	0.34	0.0074	5.88	0.31	0.0192
C5p	5.44	0.31	5.26	0.34	0.2473	5.01	0.28	0.0071
L1a	6.29	0.47	6.20	0.54	0.7010	6.13	0.55	0.5247
L2a	9.13	0.59	9.07	0.76	0.8415	9.08	0.76	0.8653
L3a	9.98	0.56	9.93	0.74	0.8712	10.07	0.75	0.7837
L4a	9.24	0.50	9.27	0.87	0.9316	9.25	0.76	0.9870
L5a	7.27	0.49	7.14	0.63	0.6277	7.19	0.58	0.7691
L1b	5.99	0.34	5.51	0.37	0.0098	5.53	0.31	0.0071
L2b	7.19	0.43	6.85	0.49	0.1350	6.97	0.48	0.3124
L3b	7.89	0.53	7.63	0.59	0.3458	7.72	0.56	0.5116
L4b	7.38	0.52	7.12	0.56	0.3270	7.13	0.59	0.3534
L5b	5.84	0.47	5.55	0.52	0.2272	5.65	0.49	0.4193

The mean and standard deviation of AE and the mean of APE were determined and the accuracy of the measurements was confirmed (Table 5). The mean AE for 10 subjects was very low both for CT scanning (0.29 ± 0.10 mm) and for SL scanning (0.30 ± 0.11 mm). The maximum AE and APE was 0.93 mm, 4.69 ± 2.33% and 0.94 mm, 4.88 ± 2.22% for CT scanning and SL scanning respectively.

**Table 5:** Absolute error (AE) and Absolute percentage error (APE) of the measuring techniques.

Measurements	CT scanning			SL scanning		
	AE (mm)	SD (mm)	APE (%)	AE (mm)	SD (mm)	APE (%)
C1i	0.15	0.11	2.34	0.40	0.26	6.14
C2d	0.26	0.11	4.98	0.48	0.17	9.00
C3d	0.25	0.12	4.70	0.48	0.23	8.93
C4d	0.41	0.18	9.25	0.25	0.17	5.65
C5d	0.35	0.18	8.54	0.19	0.11	4.51
C2p	0.17	0.14	2.85	0.18	0.16	3.03
C3p	0.14	0.13	2.26	0.18	0.13	2.91
C4p	0.50	0.13	9.10	0.41	0.23	7.44
C5p	0.27	0.12	4.91	0.43	0.18	7.85
L1a	0.17	0.08	2.66	0.16	0.13	2.50
L2a	0.26	0.16	2.84	0.27	0.14	2.92
L3a	0.20	0.15	1.95	0.24	0.15	2.37
L4a	0.39	0.13	4.25	0.28	0.14	3.02
L5a	0.25	0.23	3.49	0.21	0.16	2.92
L1b	0.50	0.29	8.26	0.46	0.20	7.71
L2b	0.34	0.13	4.76	0.33	0.13	4.58
L3b	0.26	0.18	3.30	0.30	0.15	3.83
L4b	0.26	0.14	3.58	0.31	0.24	4.21
L5b	0.29	0.13	5.00	0.19	0.16	3.22

## Discussion

To capture accurate 3D hand image rapidly with a full 360∘, we improved the design of structured light-scanning equipment. The Gemini structured-light scanning system is equipped with two modular units and allows for almost simultaneous capturing images of dorsal and palmar sides of hand. The design of double modular units is aimed to reduce experimental errors caused by the instability of the object. The system not only successfully established a complete and smooth 3D hand photograph, but also shortened the scanning and reconstruction time. The skin texture of the actual hand by SL scanning was also very delicate.

In this study, we investigated the reproducibility and accuracy of two 3D imaging techniques. To the best of our knowledge, this is the first study to compare SL scanning and CT scanning for their efficacy in 3D imaging of hand. Gemini structured-light scanning system is a new method. CT scanning is a routine technique used for diagnosis, reconstruction of soft tissue and planning of operation. Tan et al. introduced computed tomography angiography (CTA) for preoperative planning in complex toe-to-hand reconstruction^21^. In our team, CTA is also commonly used to map donor site and recipient vasculature^22–24^, and CT data were used to generate 3D models of complex soft tissue defect for individual design for perforator flap.

Three methods all showed high levels of intra-observer reliability: ICC; 0.925–0.995 for DM, ICC; 0.946–1.000 for CT scanning, and ICC; 0.984–1.000 for SL scanning. The plaster hand models instead of actual hand is one of the major reasons of high reliability. As the plaster hand models were rigid and fixed in shape, the influence of hand posture and movement on the results of hand measurement could be neglected. It may also prevent errors due to skin deformity associated with the use of the actual hand in DM. The reliability of two 3D measurements was greater than that achieved with DM. The tightness of the tape and the direction of the sliding caliper were subjective factors that might influence the values obtained in DM.

In this study, we evaluated the accuracy of Gemini structured-light scanning system in comparison with direct measurement and used the standard mentioned by Soghyia et al., i.e. that the measurement differences of more than 0.5 mm between any of the techniques were considered clinically significant^25^. Significant differences were observed in 3 of 19(15.8%) measurements. The mean AE and APE were 0.30 ± 0.11 mm and 4.88 ± 2.22% respectively in SL scanning. In 2003, Enciso and co-workers showed that a single-camera system had errors in the range of 0.48–1.55 mm^26^. Yu et al. showed the differences in the dimensions of interphalangeal finger joint circumference obtained by 3D image combined from ten captures and direct measurement yielded root mean square error (RMSE) of 1.97–5.07 mm^1^. The maximum AE obtained with Gemini structured-light scanning was 0.94 mm. This finding is comparable with the results of other studies on the accuracy of structured-light scanning applications^27^, and other imaging applications used for obtaining measurements of the hand^1,28^. Although the Gemini structured-light scanning system has not been employed for the clinical measurement of hand, the results of our study are encouraging.

The AE for the PIP circumference of the 4th finger and 1st finger length on the palmar side was greater than the value set for clinically significant difference between direct measurement and CT scanning, i.e., 0.5 mm. The mean AE of ten subjects was very low for the CT scanning(0.29 ± 0.10 mm) and for SL scanning (0.30 ± 0.11 mm). However, the mean APE of the five measurements (26.32%) obtained by CT scan and seven measurements (26.84%) obtained by the structured-light scan were greater than 5%. Fourie et al. evaluated the accuracy of standard anthropometric linear measurements of the head with three different 3D scanning systems and reported a mean AE of 0.76–0.89 mm and mean APE of 1.21–1.64% for the 3D scanning systems^9^. Although it appeared that the AE was lower in our study, the APE was much lower in the study by Fourie et al., considering that the dimensions of the head are greater than those of the hand. The 3D scanning system used for hand measurement is technically more precise than that required for head^24^.

The SL scanning offers several advantages, including rapid capture, photorealistic appearance, non-invasive nature, varying resolution quality, and high sensitivity of the technique. These advantages allow more rapid and non-invasive evaluation of the soft tissues of hand than conventional anthropometric techniques. However, one of the drawbacks of this method is the image defects caused by light irradiation and occlusion. This problem may be overcome by adding scanning heads^29^, or modifying the precise encoding and decoding algorithm of structured light^30^. However, due to inherent defects in the technology and distortion of light, none of the 3D imaging systems currently in use are accurate across the full field of view. Continuous improvement in technology and software has made it possible for researchers and clinicians to achieve realistic 3D imaging, although it may never be fully attainable. Furthermore, Lee et al. reported that subjective satisfaction achieved with the 3D semiautomatic measurement protocol (3D-SAMP) with respect to ease of hand measurement was significantly greater than that achieved for the direct measurement protocol^28^. This made the 3D-SAMP more preferable to direct measurement because of the shorter interaction time with the subject.

This study has a few limitations. First, the investigations were made in a small sample population. Second, we used plaster hand models instead of actual hands to minimize experimental errors. Yu et al.^1^ also explained that actual hands and plaster hands could differ in dimensions because of posture changes, muscular movements, and tissue compression in actual hands. Our study revealed that 3D images acquired by Gemini Structured light-scanning system and CT scanning were highly accurate. CT scanning is commonly used to harvest the static data to diagnose, reconstruct soft tissue. A real-time measurement of hand model and hand tracking is the tendency, and we intend to obtain hand measurements from live models to evaluate the hand contour and function, assess the hand/finger defect, and guide hand operation by Gemini Structured light-scanning system.

## Conclusions

The Gemini structured-light scanning system not only successfully established a complete and smooth 3D hand photograph, but also shortened the scanning and reconstruction time. We compared two 3D measurement techniques with DM, and the results indicate that in terms of accuracy, the 3D imaging measurements obtained using the two indirect hand measurement techniques did not differ from those obtained by DM.
